# Radiographic Features of Facial Cosmetic Material: Report of Two Cases

**DOI:** 10.1155/2021/7308636

**Published:** 2021-09-30

**Authors:** Noura A. Alsufyani, Mohammed A. Alsufyani

**Affiliations:** ^1^Oral & Maxillofacial Radiology, Oral Medicine and Diagnostic Sciences Department, College of Dentistry, King Saud University, Riyadh, Saudi Arabia; ^2^School of Dentistry, Department of Medicine and Dentistry, University of Alberta, Canada; ^3^Cosmetic and Dermatologic Surgery, Department of Dermatology and Dermatologic Surgery, Prince Sultan Military Medical City, Riyadh, Saudi Arabia

## Abstract

Recently, the use of noninvasive facial cosmetic procedures has been widely disseminated. In the face, cosmetic fillers, threads, and implants are used to reduce or delay the effects of aging or adjust facial defects caused by trauma or disease. The dentist or dental specialist may encounter these materials in the radiographic images of their patients. There are few reports in the dental literature describing the radiographic appearance of some materials along with the diseases they mimic. As the procedures and materials advance and evolve, dentists and dental specialists must be aware of their radiographic appearance to avoid errors in diagnosis. This is a report of two cases that include panoramic radiography and CBCT scan. Among these cases, there is an unusual appearance of a cosmetic filler due to a subperiosteal injection method. Moreover, it will discuss common types of cosmetic materials used in the face and their imaginological appearance. This is the key to dentists and dental specialists due to increasing use of facial cosmetic materials and a parallel increase in the use of cone beam CT and chances to encounter such findings.

## 1. Introduction

With aging, changes occur in various tissues of the face resulting in cutaneous changes, volume depletion, and sagging. Fillers, autologous fat, and surgical implants are used for therapeutic or cosmetic volume correction. Suspension or lifting and threads are used to overcome the sagging and laxity. According to the American Society for Aesthetic Plastic Surgery 2018, hyaluronic acid (HA) fillers have become the second most nonsurgical procedure with a 58% increase since 2014 [1]. In 2018, the American Society for Dermatologic Surgery reports 1.6 million soft tissue filler procedures, a 78% increase since 2012, and approximately 32,000 thread lift procedures [2].

Cone beam CT has become a common diagnostic tool in dentistry; however, incidental findings pose a diagnostic challenge to dentists and dental specialists. Recently, dentists showed interest and engagement in cosmetic dermatology practices such as botulinum toxin and cosmetic filler injections. In the dental literature, several case reports presented the imaginological appearance of calcium hydroxyapatite dermal fillers, cheek and chin implants, and gold threads [3–7].

This work presents the radiographic appearance of two cases of cosmetic materials, with one unusual subperiosteal filler injection, incidentally observed in cone beam CT images. With the recent crossover from dentistry to cosmetic dermatology, this work reviews the pertinent literature of cosmetic materials that can be encountered by the dentist/dental specialist in the facial area.

## 2. Case Reports

This project was approved by the Health Research Ethics Board at the University of Alberta, Canada (#00101635), and has therefore been performed in accordance with the ethical standards laid down in an appropriate version of the Declaration of Helsinki (as revised in Brazil 2013). The CBCT examination was completed at the cone beam CT services at the School of Dentistry, University of Alberta. For both cases, the i-CAT Next Generation Cone Beam (Imaging Sciences International, Hatfield, PA, USA) was used with the following protocol: 120 kVp, 20 mAs, 16 × 13 mm field of view, and 0.3 mm voxel.

### 2.1. Case 1

**Figure 1:**
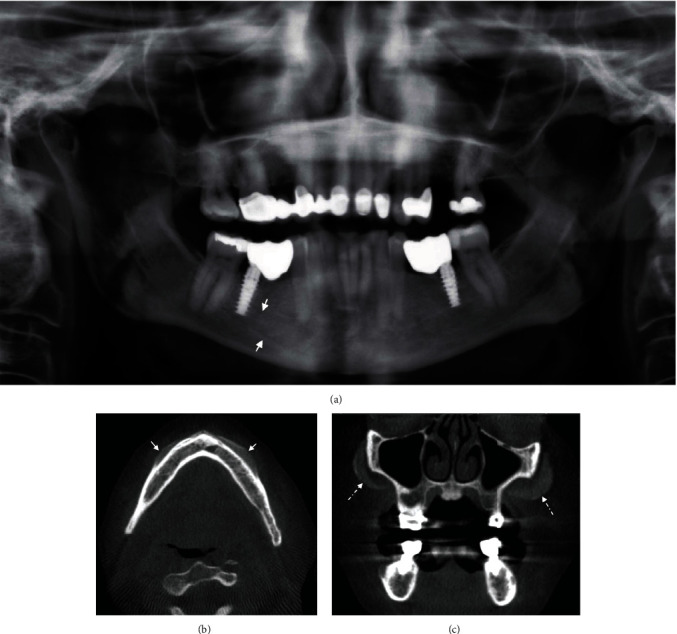
Subperiosteal cosmetic filler in the mandible (case 1): (a) panoramic radiograph showing linear radiopaque lines (arrows); (b) axial CBCT image showing a bilateral, subperiosteal cosmetic filler at the buccal cortex of the posterior mandible; (c) coronal CBCT image showing bilateral zygomatic implants (dotted arrows).

A 66-year-old female presented for maxillary and mandibular dental implant assessment. The referring clinic provided a panoramic radiography before the cone beam CT scan. On the panoramic radiograph, there were two, subtle, linear radiopaque lines extending from the apical end of implants #36 and #46 to the root apices of teeth #34 and #34, respectively, with parallel lines diverging medially and inferiorly toward the inferior border of the parasymphysis (Figure 1(a)).

The cone beam CT depicted a well-defined predominantly radiolucent entity along the buccal cortex of the mandible extending from the right mandibular molar area and crossing the midline to the left mandibular molar area (Figure 1(b)). There are few minute calcifications similar to cortical bone in density within the radiolucency. The mixed entity appears as a “subperiosteal” finding causing cortical expansion of the buccal cortex along the crestal and midalveolar levels at its distal ends and along the inferior levels at the medial ends, i.e., symphysis and parasymphysis.

In addition, there were bilateral, uniform, high-attenuation objects within the soft tissues along the outer cortex of the zygomatic process of maxilla and zygomatic bone (Figure 1(c)). The objects were fine-granular, less than cancellous bone in density. The osseous and soft tissues adjacent to these entities were unremarkable. The diagnosis was cosmetic implants of the zygoma. No differential diagnosis is offered.

The radiographic features of periphery and expansion depicted by the subperiosteal filler in the mandible based on the panoramic radiograph would suggest a benign-cystic lesion. With the presence of minute calcifications, pathological entities such as calcifying odontogenic epithelial tumor, odontogenic keratocyst with dystrophic calcifications, calcifying odontogenic cyst, and adenomatoid odontogenic tumor can be considered. The cone beam CT images revealed the uniform and symmetric shape, unusual subperiosteal location, and lack of effects on the surrounding teeth. As such, a cosmetic procedure that involves lifting the periosteum was considered rather than odontogenic pathology. The patient was contacted and has confirmed receiving cosmetic implants and fillers in the midface, mandible, and chin. A dermatology consultant confirmed that subperiosteal techniques are used in the maxillofacial and mandibular areas to deliver cosmetic fillers. Based on this information, we concluded that the entity in the mandible was a subperiosteal cosmetic filler.

### 2.2. Case 2

**Figure 2:**
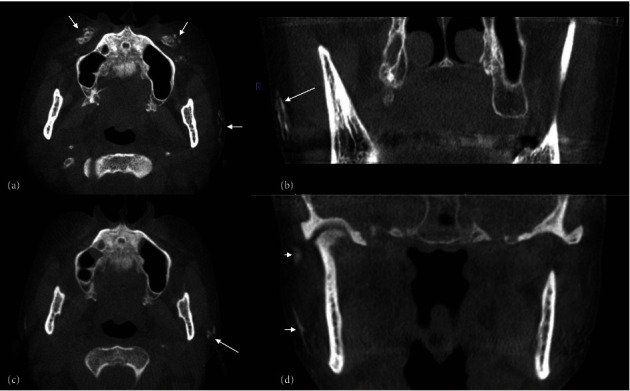
CBCT images of the subdermal CaHA filler (case 2): (a) axial and (b) coronal CBCT images showing multiple, conglomerate high-attenuation entities within the dermal layers anterior to the maxillae and lateral to the masseter muscles; (c) axial and (d) coronal CBCT images after 9 months showing resorption and posterior migration of the filler.

A 52-year-old female underwent a cone beam CT scan for preimplant and temporomandibular joint assessment. A cluster of multiple radiopacities was incidentally noted in the immediate soft tissues lateral to the left masseter muscle and bilaterally below the zygoma (Figures 2(a) and 2(b). The radiopacities were irregular in shape and similar to those in cortical and cancellous bone in density. Differential diagnoses considered osteoma cutis, myositis ossificans, heterotrophic/dystrophic calcifications, and foreign bodies. The patient was contacted and has confirmed receiving cosmetic fillers in the face. Nine months postbone graft for dental implant purposes, the filler material showed posterior-superior migration and reduction in size and density (Figures 2(c) and 2(d)).

## 3. Discussion

### 3.1. Soft Tissue Augmentation (Fillers)

Fillers are used for a wide range of indications: correcting age-related volume loss and sagging and correction of soft tissue defects caused by trauma or disease.

Fillers used for soft tissue augmentation can be synthetic such as collagen, hyaluronic acid gel, calcium hydroxyapatite (CaHA), and poly-L-lactic acid (PLLA). These are known as temporary or semipermanent due to their biodegradability. Permanent synthetic products are polymethylmethacrylate (PMMA), polyalkylimide, and liquid silicone. The source can be as well a natural autologous one as in the case of autologous fat transfer [8–11].

The level of placement of such products depends largely on the indication intended to be addressed and facial anatomy of the target area, as well as the type of product and its approved indications. Fillers are placed intradermally, immediately subdermally, or in the subcutaneous plane [12, 13].

Injectable cosmetic fillers can have radiolucency/soft tissue density (e.g., hyaluronic acid, collagen, and fat) or varying degrees of radiopacity (e.g., calcium hydroxyapatite (CHA) and silicone).

Radiopaque fillers can be depicted on plain radiographs such as panoramic radiographs but are certainly better detailed in CT and CBCT according to several case reports in the medical and dental fields [3–7]. An example of the CaHA filler is presented in Figure 2.

Less commonly, cosmetic fillers can be injected subperiosteally, such as in case 1 (Figure 1). This method of placement showed a promising technique in the midface and chin. Less postoperative complications and desirable aesthetic outcomes partly are due to creating a skeletal support for the overlaying soft tissues thus reducing drooping or sagging [14–17].

Case 1 is the first to describe the radiographic presentation of a subperiosteal filler in the mandible. The differential diagnoses of subperiosteal fillers include intraosseous tumors or cysts rather than soft tissue lesions for dermal or subcutaneous fillers. Imaging with cone beam CT offered key features for diagnosis such as the symmetrical distribution (the extension to the left mandible was not clear in the panoramic radiograph), uniform geometry in *X*, *Y*, and *Z* planes, and confinement to the subperiosteal layer. The correlation with patient history to confirm cosmetic procedures was important to reach a diagnosis without surgical intervention (surgical exploration or biopsy). Patients can be reluctant to admit to cosmetic work in their initial visit to the dentist/dental specialist. The role of the oral and maxillofacial radiologist to view, analyze, investigate, and diagnose incidental findings is emphasized in case 1.

There are no direct dental implications for the cosmetic filler; however, the dental clinician must be aware of their existence and possible complications. The most common complications of fillers are transient and mild in nature such as swelling, erythema, and bruising. Other, less common, complications include infection, noninflamed nodules, biofilm, granuloma, and persistent edema (i.e., malar). And similar to case 2 (Figure 2), migration of the filler material has been reported [9, 11, 18].

### 3.2. Threads for Lifting

Threads are minimally invasive methods to lift saggy skin that results from increased laxity and give a more youthful appearance. Common types of threads used are polypropylene suture, poly-L-lactic acid (PLLA), polydioxanone (PDO), and polylactide/glycolide resorbable copolymer (PLGA) [19–21]. Gold thread therapy involves implanting pure gold threads of 0.1 mm diameter braided with polyglycolic acid or polyvinylidene difluoride into the subdermal skin. The foreign-body reaction of this therapy may trigger the production of elastin and collagen fibers and provide mechanical support to the tissues and ultimately skin rejuvenation [22, 23]. However, there is little evidence for its therapeutic efficacy [23–25].

Threads have either barbs or cons fixated on their surfaces. After marking the overlying skin with the best oriented vector to give the maximum lift effect, the threads are placed in the fibrous fatty tissue in the subcutaneous plane to lift and fix the tissue in their central part without the need to anchor either ends [20, 21, 26].

Most threads are radiolucent unless braided with gold. Tanaka Kikinzoku Kogyo KK (Tokyo, Japan) originally coated nonabsorbable cog thread with 24-carat gold [27]. Despite some commercial reports about the rejuvenation provided by gold threads, no scientific reports exist on their use except for a histological study on rats suggesting “that the gold-coated cog thread has clinical potential.” [27]

Gold threads can be depicted in panoramic radiographs, in CBCT images, or in periapical radiographs if the cheek intercepts the path of the X-ray beam [23–25]. The differential diagnosis may include acupuncture needles, surgical clips, and posttrauma foreign bodies.

The cosmetic or acupuncture gold wires may cause radiographic artifacts and with time become fragmented and unevenly distributed [24, 25]. Complications that can occur with thread lifting are usually mild and temporary such as swelling, bruising, erythema, and skin dimpling. Less common complications are infection, foreign body reaction, hematoma, thread migration, and exposure [19, 21].

### 3.3. Surgical Implants

Implants have been used to correct deformities (congenital or acquired) and changes resulting from facial aging. Facial regions most treated are the dorsum of the nose, malar area (Figure 1), and the chin.

There are different types of implants and depending on their composition can be radiolucent (Permacol™, MERSILENE® resorbable mesh, LactoSorb®, MEDPOR®, methyl methacrylate, polyethylene, etc.) or present with a degree of radiopacity (autologous bone graft, Silastic™ silicone, titanium wire, polytetrafluoroethylene, etc.) [27, 28]. These implants need fixation by using screws or sutures or through creating pockets and contouring done at the subperiosteal plane to be immobilized [27].

Complications that may occur with placing facial implants include infection, displacement, hematoma, damage to the infraorbital or mental nerves, bone resorption, atrophy of the overlying skin, and extrusion [27, 28].

## 4. Conclusion

With the exponential demand of cosmetic procedures in the face, the dentists and dental specialists are bound to encounter these materials in conventional radiographs or cone beam CT images. Imaginological appearance varies widely in terms of shape and radiodensity depending on the material used. Recognizing these cosmetic materials and their imaginological appearance will improve diagnosis, avoid mismanagement or unnecessary biopsy, and anticipate any future complications of the material in the mandibular and maxillofacial area.

## Data Availability

Data is available upon reasonable request.

## References

[1] American society for aesthetic plastic surgery 2018 statistics. https://www.surgery.org/consumers/procedures.

[2] American society for dermatologic surgery 2018 statistics. https://www.asds.net.

[3] Cabrera M. A., Mulinari-Brenner F. (2011). Radiological evaluation of calcium hydroxyapatite-based cutaneous fillers. *Surgical and Cosmetic Dermatology*.

[4] Koka S., Shah K., Mallya S. (2017). Dermal filler presenting as lobular radiopacities in an edentulous patient: a clinical report. *Journal of Prosthodontics*.

[5] Kwon Y., An C., Choi K. S., Lee D., An S. (2018). Radiographic study of dermal fillers in the facial area: a series of 3 cases. *Imaging Science in Dentistry*.

[6] Mundada P., Kohler R., Boudabbous S., Toutous Trellu L., Platon A., Becker M. (2017). Injectable facial fillers: imaging features, complications, and diagnostic pitfalls at MRI and PET CT. *Insights into imaging*.

[7] Valiyaparambil J. R., Rengasamy K., Mallya S. M. (2009). An unusual soft tissue radiopacity - radiographic appearance of a dermal filler. *British Dental Journal*.

[8] Kim J. J., Evans G. R. D. (2012). Applications of biomaterials in plastic surgery. *Clinics in Plastic Surgery*.

[9] Carruthers J., Carruthers A., Humphrey S. (2015). Introduction to fillers. *Plastic and Reconstructive Surgery*.

[10] Woodward J., Khan T., Martin J. (2015). Facial filler complications. *Facial Plastic Surgery Clinics of North America.*.

[11] Chiang Y. Z., Pierone G., Al-Niaimi F. (2017). Dermal fillers: pathophysiology, prevention and treatment of complications. *JEADV*.

[12] Mckee D., Remington K., Swift A., Lambros V., Comstock J., Lalonde D. (2019). Effective rejuvenation with hyaluronic acid fillers: current advanced concepts. *Plastic and Reconstructive Surgery*.

[13] Surek C. C. (2019). Facial anatomy for filler injection: the superficial musculoaponeurotic system (SMAS) is not just for facelifting. *Clinics in Plastic Surgery*.

[14] Martínez-Sanz E., Ossipov D. A., Hilborn J., Larsson S., Jonsson K. B., Varghese O. P. (2011). Bone reservoir: injectable hyaluronic acid hydrogel for minimal invasive bone augmentation. *Journal of Controlled Release*.

[15] Pavicic T., Yankova M., Schenck T. L. (2020). Subperiosteal injections during facial soft tissue filler injections is it possible?. *Journal of Cosmetic Dermatology*.

[16] Heffelfinger R. N., Blackwell K. E., Rawnsley J., Keller G. S. (2007). A simplified approach to midface aging. *Archives of Facial Plastic Surgery*.

[17] Kang K., Chai C. (2017). Subperiosteal chin augmentation with hyaluronic acid filler in patients with a small chin. *Journal of Cosmetic Medicine*.

[18] Kontis T. C., Bunin L., Fitzgerald R. (2018). Injectable fillers: panel discussion, controversies, and techniques. *Facial Plastic Surgery Clinics of North America*.

[19] Gülbitti H. A., Colebunders B., Pirayesh A., Bertossi D., van der Lei B. (2018). Thread-lift sutures: still in the lift? A systematic review of the literature. *Plastic and Reconstructive Surgery*.

[20] Archer K. A., Garcia R. E. (2019). Silhouette instalift: benefits to a facial plastic surgery practice. *Facial Plastic Surgery Clinics of North America*.

[21] Atiyeh B. S., Dibo S. A., Costagliola M., Hayek S. N. (2010). Barbed sutures “lunch time” lifting: evidence-based efficacy. *Journal of Cosmetic Dermatology*.

[22] Shin K. C., Bae T. H., Kim W. S., Kim H. K. (2012). Usefulness of gold thread implantation for crow's feet. *Archives of Plastic Surgery*.

[23] Alsaadi G., Jacobs R., Quirynen M., van Steenberghe D. (2008). Soft tissue augmentation of the cheeks detected on intra- and extraoral radiographs: a case report. *Dentomaxillofacial Radiology*.

[24] Negayama R., Fujikawa T. (2018). CT appearance of gold thread facelift. *QJM*.

[25] Keestra J. A. J., Jacobs R., Quirynen M. (2014). Gold-wire artifacts on diagnostic radiographs: a case report. *Imaging Science in Dentistry*.

[26] Villa M. T., White L. E., Alam M., Yoo S. S., Walton R. L. (2008). Barbed sutures: a review of the literature. *Plastic and Reconstructive Surgery*.

[27] Oliver J. D., Eells A. C., Saba E. S. (2019). Alloplastic facial implants: a systematic review and meta-analysis on outcomes and uses in aesthetic and reconstructive plastic surgery. *Aesthetic Plastic Surgery*.

[28] Schwartz D., Quereshy F. A. (2014). Combined rhytidectomy and alloplastic facial implants. *Atlas of the Oral and Maxillofacial Surgery Clinics of North America*.

